# Classification and Regression Tree and Spatial Analyses Reveal Geographic Heterogeneity in Genome Wide Linkage Study of Indian Visceral Leishmaniasis

**DOI:** 10.1371/journal.pone.0015807

**Published:** 2010-12-31

**Authors:** Michaela Fakiola, Anshuman Mishra, Madhukar Rai, Shri Prakash Singh, Rebecca A. O'Leary, Stephen Ball, Richard W. Francis, Martin J. Firth, Ben T. Radford, E. Nancy Miller, Shyam Sundar, Jenefer M. Blackwell

**Affiliations:** 1 Cambridge Institute for Medical Research and Department of Medicine, University of Cambridge School of Clinical Medicine, Cambridge, United Kingdom; 2 Institute of Medical Sciences, Banaras Hindu University, Varanasi, India; 3 Telethon Institute for Child Health Research, Centre for Child Health Research, The University of Western Australia, Subiaco, Australia; 4 Australian Institute of Marine Science, The UWA Oceans Institute, The University of Western Australia, Crawley, Australia; University of Texas MD Anderson Cancer Center, United States of America

## Abstract

**Background:**

Genome wide linkage studies (GWLS) have provided evidence for loci controlling visceral leishmaniasis on Chromosomes 1p22, 6q27, 22q12 in Sudan and 6q27, 9p21, 17q11-q21 in Brazil. Genome wide studies from the major focus of disease in India have not previously been reported.

**Methods and Findings:**

We undertook a GWLS in India in which a primary ∼10 cM (515 microsatellites) scan was carried out in 58 multicase pedigrees (74 nuclear families; 176 affected, 353 total individuals) and replication sought in 79 pedigrees (102 nuclear families; 218 affected, 473 total individuals). The primary scan provided evidence (≥2 adjacent markers allele-sharing LOD≥0.59; nominal *P*≤0.05) for linkage on Chromosomes 2, 5, 6, 7, 8, 10, 11, 20 and X, with peaks at 6p25.3-p24.3 and 8p23.1-p21.3 contributed to largely by 31 Hindu families and at Xq21.1-q26.1 by 27 Muslim families. Refined mapping confirmed linkage across all primary scan families at 2q12.2-q14.1 and 11q13.2-q23.3, but only 11q13.2-q23.3 replicated (combined LOD = 1.59; *P* = 0.0034). Linkage at 6p25.3-p24.3 and 8p23.1-p21.3, and at Xq21.1-q26.1, was confirmed by refined mapping for primary Hindu and Muslim families, respectively, but only Xq21.1-q26.1 replicated across all Muslim families (combined LOD 1.49; *P* = 0.0045). STRUCTURE and SMARTPCA did not identify population genetic substructure related to religious group. Classification and regression tree, and spatial interpolation, analyses confirm geographical heterogeneity for linkages at 6p25.3-p24.3, 8p23.1-p21.3 and Xq21.1-q26.1, with specific clusters of families contributing LOD scores of 2.13 (*P* = 0.0009), 1.75 (*P* = 0.002) and 1.84 (P = 0.001), respectively.

**Conclusions:**

GWLS has identified novel loci that show geographical heterogeneity in their influence on susceptibility to VL in India.

## Introduction

Visceral leishmaniasis (VL) is a parasitic disease primarily caused by the *Leishmania donovani* species complex (*L. d. donovani*, *L. d. infantum*/*chagasi*, and *L. d. archibaldi*). Ninety percent of the (under)-estimated 500,000 annual new cases occur in India/Bangladesh/Nepal, Brazil, and Sudan (WHO statistics on leishmaniasis http://www.who.int/topics/leishmaniasis/en/). Although only 10–20% of infected individuals develop disease, clinical VL is fatal if left untreated. The remaining 80–90% of human infections is sub-clinical or asymptomatic, usually associated with strong cell-mediated immunity to *Leishmania* antigen [1]–[4].

Genetic susceptibility to VL was initially demonstrated in mice [5]. In humans, familial clustering [6], ethnic differences [7]–[9], and high relative risk (λ_2_ = 34) of disease in further siblings of affected sibling pairs [10] support a role for host genotype in determining disease outcome. Genetic studies in endemic populations from Brazil and Sudan provide evidence for a number of candidate genes [11]–[13] and chromosomal regions [14]–[17] controlling susceptibility and resistance to VL.

Family-based genome-wide linkage studies (GWLS) have been used to identify novel regions of the genome linked to disease susceptibility. Bucheton and coworkers [14] first reported genome-wide significance (LOD>3.6, nominal *P*≤2×10^−5^; following Lander and Kruglyak [18]) for a major gene controlling VL susceptibility on chromosome 22q12 (LOD score 3.5; nominal *P* = 3×10^−5^) in a village near Gedaref in Eastern Sudan occupied by the Aringa ethnic group. Miller et al. [17] similarly reported genome-wide significance for Y-chromosome lineage and village-specific susceptibility loci at Chromosomes 1p22 (1p22, LOD = 5.65; nominal *P* = 1.72×10^−7^) and 6q27 (6q27, LOD = 3.74; nominal *P* = 1.68×10^−5^) for two villages, located ∼40 kilometers (km) apart and 100 km south of Gedaref, that are occupied by members of the related Masalit ethnic group. It was proposed that these major differences over a small geographical range were due to founder effect and consanguinity in recently immigrant populations. GWLS conducted in Brazil also provide positive evidence (LOD≥1.1, nominal *P*-values ≤0.01) for loci on Chromosomes 6q27, 7q11.22, and 17q11.2-q21.3 [15], and on 9q [16].

India accounts for up to 50% of VL globally, with adverse effects linked to socio-economic status of its population [19], [20]. Genetic susceptibility in this major focus for VL has not been investigated to date. Here we present results of a GWLS conducted in a rural population from Bihar State, the most affected state in India where the 1991–1992 epidemic resulted in 250,000 cases [19]. The results provide positive evidence for novel chromosomal regions linked to disease susceptibility that show local geographical heterogeneity across this region of India that may be contributed to by familial clustering and consanguinity.

## Methods

### Study site

In India, anthroponotic clinical VL is also known as kala-azar. Our study sample comprises Indian multicase families with clinical VL derived from villages located within a radius of ∼60 km from the city of Muzaffarpur covering the districts of Muzaffarpur, Vaishali, and Sitamarhi. Global positioning system (GPS) coordinates for each family were collected to facilitate spatial analysis. Further epidemiological and demographic details relating to the study site are described elsewhere [20]. An annual incidence rate of 2.49 clinical VL cases/1,000 persons has been reported in the region [21]. Although India is considered an endemic site for post kala-azar dermal leishmaniasis, very few cases of this form of disease were reported in this study area [22]. *L. donovani sensu strictu* (zymodeme MON-2) was confirmed as the causative agent of VL in the study region, in accordance with other reports on clinical isolates from kala-azar patients in the state of Bihar [23]–[26]. Non-invasive buccal swabs were collected and DNA prepared by whole genome amplification directly from the buccal swab buffer according to the manufacturers' instructions (Repli-g, QIAGEN). Approval for the study was provided by the Ethical Committee of the Institute of Medical Sciences, Banaras Hindu University, Varanasi, India. Informed written consent in Hindi was obtained from all participating individuals and from parents of children under 18 years old.

### Diagnosis

Families with at least two siblings affected with clinical VL were ascertained from medical records held in the Kala-Azar Medical Research Centre (KAMRC) in Muzaffarpur, India. Diagnosis of VL was based on presence of typical clinical features of VL i.e. fever with rigors and chills, splenomegaly, weight loss and pancytopenia followed by demonstration of parasites by parasitological methods (light microscopy, *in vitro* culture) using splenic aspirates [27]. Additional VL cases identified in the field were confirmed on the basis of proof of medical records of diagnosis and treatment issued from one of the local health clinics or private practice, and accompanied by demonstration of clinical response to anti-leishmanial treatment (typically with amphotericin B).

### Families

In total, 58 clinical VL multicase pedigrees (74 nuclear families; 147 affected offspring; 353 individuals) (Table 1), comprising 31 Hindu and 27 Muslim pedigrees, were used in the primary GWLS and refined mapping. A further 79 pedigrees (102 nuclear families; 166 affected offspring; 473 individuals), collected from the same study area, were used for replication analysis. The 137 families (Table 1) included 238 affected males (mean ± SD age at diagnosis 17.39±12.85 years; range 1–67 years) and 156 affected females (mean ± SD age at diagnosis 15.78±13.42 years; range 1–62 years). The affected parents reported in Table 1 were all historical cases >30 years of age at the time of sample collection. The 1.5-fold more male than female cases of clinical VL in the pedigrees is in concordance with the reported increased risk of VL in males in this region of India [19], [28].

**Table 1:** Family structure and distribution by religious group of the Indian primary genome scan and additional clinical VL multicase families.

Family Structure	Primary Families			Additional Families		
	Hindu	Muslim	Total	Hindu	Muslim	Total
N^o^ families	31	27	58	52	27	79
N^o^ nuclear families	39	35	74	69	33	102
Nuclear families with 1 affected sib^a^	10	6	16	33	14	47
Nuclear families with 2 affected sibs	24	23	47	30	18	48
Nuclear families with 3 affected sibs	3	5	8	5	1	6
Nuclear families with 4 affected sibs	2	0	2	0	0	0
Nuclear families with 5 affected sibs	0	1	1	1	0	1
N^o^ affected offspring	75	72	147	113	53	166
N^o^ affected parents	12	13	25	27	11	38
Total N^o^ affected individuals	89	87	176	151	67	218
Total N^o^ individuals	181	172	353	333	140	473

Numbers are given for successfully genotyped individuals.

a All nuclear families with one affected offspring were part of a larger multicase pedigree.

### Genotyping

Genotyping of the primary genome scan families was performed by deCODE genetics (Reykjavík, Iceland) for 515 polymorphic markers covering all 22 autosomes and the X chromosome. The average interspacing between markers was 10cM according to the deCODE Haldane genetic positions. The overall rate of missing genotypes was 9%. We also genotyped 17 additional polymorphic markers (Applied Biosystems) to produce a 2-3cM refined map of the primary scan positive regions. The 79 replication families were genotyped for positive markers from the original deCODE panels, as well as the 17 additional markers, as indicated. The integrity of all families was assessed using the PedCheck v1.1 software [29]. The information provided in Table 1 represents the family sample available after checking for genetic integrity.

### Genetic statistical analyses

Allele data for all microsatellites were entered in the Genetic Information Environment (GenIE) database, developed in house (Francis R. *et al.* manuscript in preparation), and data files outputted in formats appropriate for analyses in SPLINK [30] or ALLEGRO [31]. Allele frequencies at marker loci were calculated in SPLINK [30] for all unrelated individuals in the pedigrees. Hardy-Weinberg Equilibrium (HWE) proportions for unrelated unaffected individuals in the populations were checked using the GenAssoc package (available at http://www-gene.cimr.cam.ac.uk/clayton/software/stata/) implemented within STATA v8.0. Markers that deviated from HWE (*P*<9.5×10^−5^) were excluded from analysis. Non-parametric linkage analysis was performed in ALLEGRO [31]. The S_pairs_ scoring function was used to compare allele-sharing identical-by-descent (IBD) across all affected relative pairs within the extended pedigrees, including the singleton families within larger pedigrees (Table 1) [32], [33]. Unaffected members within pedigrees were included to assist ALLEGRO to infer missing parents' genotypes. The unequal sizes of the pedigrees used in this study were taken into account by employing the *power:0.5* argument in ALLEGRO. Results are reported as allele-sharing LOD scores and plotted as sign(dhat)xLOD. All LOD scores reported are multipoint. Nominal *P*-values (i.e. without correction for multiple testing) are reported throughout. Information content for markers was estimated in ALLEGRO.

Simulations (100) performed within ALLEGRO using data for a typical set of six linked polymorphic microsatellite markers (9–14 alleles; average heterozygosity 0.7) showed that the primary genome scan family set (Table 1) across both religious groups had 95–100% power to detect a major gene at an allele-sharing LOD score of 4.91 (*P* = 1×10^−6^). The separate Hindu and Muslim family sets had >89% and >94% power to detect a major gene at an allele-sharing LOD score value of 3 (*P* = 1×10^−4^) and 2.07 (*P* = 1×10^−3^), respectively. The power within the additional families to detect a major gene at an allele-sharing LOD score of 3 (*P* = 1×10^−4^) was 63% in the Muslim families, and 100% in the Hindu families and across all additional families.

The Pedigree RElationship Statistical Test (PREST) program [34] was used to estimate degree of relatedness between individuals in our families by estimating the probabilities *P*_0_, *P*_1_, and *P*_2_ of two individuals sharing 0, 1, and 2 alleles identical-by-descent over the 515 microsatellite markers successfully genotyped in the primary genome scan families. Unrelated individuals should have probabilities 1, 0, and 0, respectively. Full sibs have *P*_0_ sharing probabilities of 0.25; half sibs plus first cousin 0.375; grandparent-child, avuncular or half-sib 0.50; double first cousins 0.5625; first cousins or half-avuncular 0.75; half first cousins 0.875; and second cousins 0.9375. We therefore considered any relationship between parents in the families with *P*_0_ sharing probabilities <0.95 to be indicative of a consanguineal marriage, with predicted relationships as outlined in Table 2. This analysis revealed that 47% of parental pairs show varying degrees of relatedness consistent with the occurrence of consanguineal marriages in both religious groups. On the basis of these predicted relationships, inbreeding loops were added within each pedigree to correct [35], [36] for the possibility of type I errors during linkage analyses due to increased allele IBD sharing in the families with over-related parents.

**Table 2:** PREST analysis for misspecified parental relationships in the primary genome scan families. The number of parents sharing a range of *P*_0_ values and their likely relationship is shown.

*P*_0_ (IBD)	Relationship	Pairs of available parents			% (N = 47)
		Hindu	Muslim	Total	
*P*_0_≈1	Unrelated	15	10	25	53.2
*P*_0_≈0.935	second cousin	8	3	11	23.4
*P*_0_≈0.875	half-first cousin	2	5	7	14.9
*P*_0_≈0.75	first cousin/half avuncular	0	3	3	6.4
*P*_0_≈0.5	half-sib/grandparent-child/avuncular	0	1	1	2.1

### Substructure, classification tree and spatial analyses

The population structure of the primary scan families was inferred using the model-based clustering software STRUCTURE [37]. STRUCTURE uses multilocus genotype data to assign individuals to a user-specified number of subpopulations (K), or jointly to two or more populations. Unrelated individuals from the primary scan families were tested for K = 1 to 4 subpopulations using three different ancestry models and assuming that the allele frequencies are fairly similar across this Indian population: (i) *Admixture/Correlated frequencies*, (ii) *No admixture/Correlated frequencies* and (iii) *Linkage/Correlated frequencies*. The correct value of K is determined by the estimated posterior probabilities, for which values closer to zero indicate a better fitted model. All models were run ten times using 50,000 iterations for the burning period and 100,000 Monte Carlo Markov Chain repetitions.

Population structure was also examined by carrying out principal component analysis using the SMARTPCA within EIGENSOFT [38], [39]. Genotypes for each microsatellite locus with *n* alleles were recorded as *n*-1 biallelic loci [40]. Microsatellite loci with high missingness (>20%) were excluded from analysis.

GPS data for latitude and longitude for all CART and spatial analyses were converted to the universal transverse mercator (UTM) coordinates system. Following this system, each family is located at a point defined in terms of UTM northing meters (m) (Y-axis in Figures 3, 4 and 5; equivalent to latitude) and UTM easting m (X-axis in Figures 3, 4 and 5; equivalent to longitude) of the zero point defined for Zone 45 of the global UTM system (http://members.rediff.com/gisindia/zone_num.jpg).

To determine whether geographical location and religion were influencing linkage results, we first carried out classification and regression tree (CART) analysis implemented using the Recursive Partitioning algorithm in the RPART package [41] (Therneau TM & Atkinson EJ. 2003 The rpart package. Software manual. http://cran.r-project.org/web/packages/rpart/index.html) in R [42]. For each marker on each chromosome, regression trees were used to explore the relationship of LOD score to UTM northing and easting locations and to religion. Regression trees are binary decision trees, which are built by repeatedly splitting the predictor space according to splitting rules based on the predictor variables [43]. In contrast to linear regression, this modelling approach helps capture complex interaction. Each branching *j  =  1,…, J* of a tree separates families *i* into two branches determined by a splitting node S*_j_* defined by a splitting rule R*_j_* of a particular variable V*_j_*. For example in chromosome 6 marker D6S244 (presented below in Figure 4a), the first branch is determined by splitting the variable V_1_(*i*), UTM north for family *i*, on S_1_(*i*): V_1_(*i*) <2886078, where 2886078 is the splitting rule R*_j_* of this branch. The left branch contains cases where the decision rule evaluates to be true, that is i: S_j_(*i*)  =  T (i.e. families located UTM north <2886078), and the right branch contains the remaining families *i*: S*_j_*(*i*)  =  F (i.e. families >2886078). We denote n*_j_* as the number of families at node *j*. Using the RPART algorithm, variables are selected to maximize the difference between the left and right branches of the regression tree, according to the Gini or Information criterion [41] (Therneau TM & Atkinson EJ. 2003. The rpart package. Software manual. http://cran.r-project.org/web/packages/rpart/index.html). Cross-validation is applied in RPART to evaluate the cross-validation prediction error of the tree, and choose the tree size where this first achieves a distinct minimum. The results of the trees were then mapped spatially using ArcGIS software.

In a second and independent approach to visualizing the spatial distribution of LOD scores for markers at the peaks of linkage on Chromosomes 6 and 8 we used spatial interpolation within ArcGIS to attribute values for LOD scores at regular grid locations based on measurements taken within the same area [44]. This provided an interpolated surface of LOD for the Chromosome 6 and 8 markers across the 31 km (north-south) by 58 km (east-west) area that includes the locations of the 58 families used in the primary GWLS. For each microsatellite marker, data were interpolated at 0.5 km grid-cell resolution as a first-order inverse-distance weighted average of the 58 data points. Thus, a data point that is 2 km from the centroid of a given grid cell contributes exactly 8 times more to the cell's weighted average LOD than a data point that is 16 km from the centroid.

## Results

### The primary GWLS

**Figure 1:**
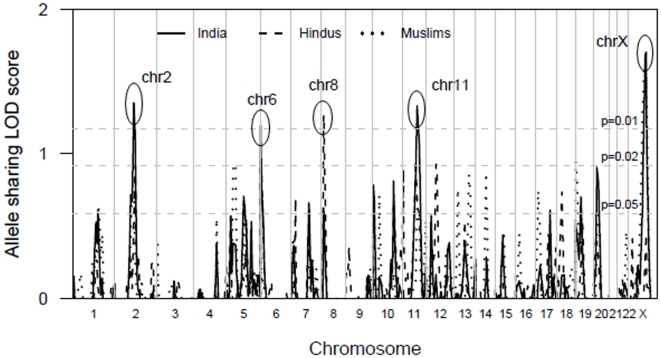
GWLS of 353 individuals from 58 Indian cVL multicase families. Multipoint allele sharing LOD scores (plotted as sign (dhat) x LOD) are shown on the vertical axis across all chromosomes from the p terminus on the horizontal axis for all Indian as well as the Hindu and Muslim religious groups. The horizontal dashed lines indicate the one-sided *P* = 0.05 and *P* = 0.01 cut offs corresponding to LOD scores of 0.58 and 1.17, respectively. Regions of linkage at *P*<0.01 are shown for chromosomes 2, 6, 8, 11 and X.

A 10cM primary GWLS of 515 polymorphic markers was carried out in 58 pedigrees (74 nuclear families). Regions of linkage were defined as having more than one marker with nominal *P*≤0.05 equating to allele-sharing LOD≥0.59. These criteria were chosen because in a true disease-susceptibility locus, adjacent markers should be correlated and share positive LOD scores. Thus observing nearby markers with low *P*-values by chance should be unlikely [45]. Non parametric linkage analysis of the primary genome scan families identified regions on chromosomes 2, 5, 6, 7, 8, 10, 11, 20 and X that met these criteria (Figure 1; solid line for all Indian primary scan families).

Indian populations are ethnically and genetically diverse [46]. Intermarriage between members of different religious groups is rare and differing levels of endogamy have been observed amongst both Hindu caste and Muslim groups [47], [48]. Studies on the genetic origins of Indian Muslims have provided contradictory results regarding the occurrence and extent of religion-driven heterogeneity in the Indian population [49]–[53]. To determine whether there was heterogeneity in our family cohort driven by genetic differences between religious groups we carried out separate linkage analyses for Hindu and Muslim families used in the primary GWLS (Figure 1; dotted line for Hindu, dashed line for Muslim). This revealed that the linkage peaks on Chromosomes 6 and 8 were largely contributed to by the 31 Hindu families studied, whereas the linkage peak on Chromosome X was largely contributed to by the 27 Muslim families studied.

Only those chromosome regions that achieved LOD≥1 (nominal *P*≤0.02) in analysis of all families and/or the religion-specific analysis were taken forward in refined mapping and replication studies.

### Refined mapping and replication studies

In the refined mapping study, 7 additional markers in positive regions of linkage on Chromosomes 2 (3 additional markers) and 11 (4 additional markers) were genotyped in all 58 primary genome scan families (Table 3). Following this grid tightening of the primary genome scan families (Table 3), the Chromosome 2q12.2-q14.1 linkage peak was retained at D2S293 (LOD 0.85; nominal *P* = 0.024), while the Chromosome 11q14.2 peak was retained and enhanced at D11S1780 (LOD 1.48; nominal *P* = 0.005). For Chromosomes 2 (7 markers) and 11 (7 markers), microsatellites that were positive for linkage in the primary scan, as well as the 7 additional microsatellites added for the refined mapping, were genotyped in the 79 additional multicase families. No evidence for linkage at Chromosome 2q12.2-q14.1 (LOD scores -0.18 to 0.08; data not otherwise shown) was observed in the additional families. Positive evidence for replication at Chromosome 11q13.2-q23.3 was observed with the peak of linkage at D11S4090 (LOD 0.67; *P* = 0.039; data not otherwise shown) ∼22cM distal to the peak observed at D11S1780 in the primary genome scan families. The combined analysis of primary and additional families for Chromosome 11 confirmed the peak of linkage at D11S4090 (LOD = 1.59; *P* = 0.0034) on Chromosome 11q23.1.

**Table 3:** ALLEGRO multipoint linkage analyses performed for the 58 primary genome scan families before and after grid tightening by genotyping of 7 additional markers on chromosomes 2 and 11.

Chromosomal Region and Marker	Distance (cM)	Primary Scan		Refined Map	
		LOD (% IC)	*P*-value	LOD (% IC)	*P*-value
**2q12.2-q14.1**					
D2S2152	0	0.25 (70)	0.142	0.32 (75)	0.111
D2S286	7.3	…		0.39 (86)	0.091
D2S2116	8.9	0.72 (71)	0.034	0.40 (88)	0.089
D2S388	18.1	0.67 (73)	0.04	0.03 (85)	0.356
D2S2216	19.2	…		0.00 (84)	0.5
D2S293	27.9	**1.35** (79)	**0.006**	**0.85** (81)	**0.024**
D2S160	32.8	…		0.74 (80)	0.032
D2S363	36.3	1.13 (75)	0.011	0.84 (81)	0.024
D2S347	43.7	0.37 (78)	0.096	0.30 (78)	0.119
D2S2271	49	0.14 (77)	0.211	0.13 (77)	0.217
**11q13.2-q23.3**					
D11S4087	0	0.50 (51)	0.065	0.38 (62)	0.093
D11S1314	4.9	…	…	0.86 (80)	0.023
D11S937	11.6	0.86 (80)	0.023	1.24 (88)	0.008
D11S901	15.2	…	…	1.34 (84)	0.007
D11S1780	20.3	**1.33** (74)	**0.007**	**1.48** (80)	**0.005**
D11S898	30.9	…	…	0.88 (82)	0.022
D11S1886	33.9	1.06 (59)	0.014	1.21 (75)	0.009
D11S4090	42.6	…	…	0.99 (82)	0.017
D11S908	44.9	0.39 (62)	0.09	0.72 (79)	0.035

Allele sharing LOD scores are reported as sign (dhat) x LOD. The highest LOD score value for each chromosome is highlighted in bold. IC  =  information content. Results of refined analysis were essentially similar when corrected for over-relatedness of parents by addition of inbreeding loops in the pedigrees on the basis of relationships determined using PREST analysis (Table 2).

For Chromosomes 6 and 8, grid tightening by genotyping of 4 additional markers was only undertaken in the 31 Hindu primary genome scan families (Table 4). Following this grid tightening, the Chromosome 6p25.3-p24.3 linkage peak was refined to the more distal location at D6S1617/D6S1574 (LOD 0.90; nominal *P* = 0.021), while the Chromosome 8p23.1 peak was refined to the closely adjacent D8S550 (LOD 1.79; nominal *P* = 0.002). For Chromosomes 6 (5 markers) and 8 (5 markers) microsatellites that were positive for linkage in the primary scan, as well as the 4 additional microsatellites added for the refined mapping, were genotyped in 52 of the additional 79 multicase families that were Hindu. No evidence for linkage at Chromosome 6p25.3-p24.3 (LOD scores -0.235 to -0.003; data not otherwise shown) or at Chromosome 8p23.1-p21.3 (LOD scores -0.298 to 0.001; data not otherwise shown) was found in these additional Hindu families.

**Table 4:** ALLEGRO multipoint linkage analyses performed for the primary genome scan families before and after grid tightening by genotyping of 10 additional markers on chromosomes 6 and 8 for 31 Hindu families and Chromosome X for 27 Muslim families.

Chromosomal region and Marker	Distance (cM)	Primary Scan		Refined Map	
		LOD (% IC)	*P*-value	LOD (% IC)	*P*-value
**6q25.3-q24.3**					
D6S244	0	**0.96** (74)	**0.018**	0.84 (76)	0.025
D6S1617	6.5	0.83 (75)	0.025	0.88 (81)	0.022
D6S1574	10.7	…	…	**0.90** (87)	**0.021**
D6S309	15.9	0.46 (72)	0.073	0.67 (79)	0.04
**8p23.1-p21.3**					
D8S503	0	…	…	0.77 (93)	0.03
D8S516	0.6	1.10 (80)	0.012	1.10 (92)	0.012
D8S520	3.8	**1.44** (79)	**0.005**	1.75 (84)	0.002
D8S550	4.9	…	…	**1.79** (87)	**0.002**
D8S552	10.3	…	…	1.11 (89)	0.012
D8S258	24.8	0.33 (74)	0.109	0.30 (76)	0.12
**Xq21.1-q26.1**					
DXS986	0	…	…	0.18 (94)	0.185
DXS1217	5.1	0.78 (90)	0.029	0.58 (96)	0.051
DXS990	10.1	…	…	0.68 (86)	0.039
DXS6799	14.7	1.19 (82)	0.010	0.97 (88)	0.018
DXS8020	17.1	1.20 (83)	0.009	1.05 (91)	0.014
DXS1106	18.7	…	…	1.13 (87)	0.011
DXS1059	23.4	…	…	0.90 (84)	0.021
DXS8055	31	1.43 (68)	**0.005**	**1.32 (75)**	**0.007**
DXS8067	36.1	…	…	0.77 (94)	0.030
DXS1001	36.7	1.34 (81)	0.007	0.80 (94)	0.027
DXS8059	40	1.36 (74)	0.006	0.91 (85)	0.020
DXS1047	48	…	…	0.35 (93)	0.103

Allele sharing LOD scores are reported as sign (dhat) x LOD. The highest LOD score value for each chromosome is highlighted in bold. IC  =  information content. Results of refined analysis were essentially the same when corrected for over-relatedness of parents by addition of inbreeding loops in the pedigrees on the basis of relationships determined using PREST analysis (Table 2).

For Chromosome X, grid tightening by genotyping of 6 additional markers was only undertaken in the 27 Muslim primary genome scan families (Table 4). The Chromosome Xq23 linkage peak was retained at DXS8055 (LOD 1.32; nominal *P* = 0.007). Six microsatellites that were positive for linkage in the primary scan, as well as the 6 additional microsatellites added for the refined mapping, were genotyped in 26 of the additional 79 multicase families that were Muslim. Borderline evidence for linkage at DXS8055 (LOD 0.40; nominal *P* = 0.088; data not otherwise shown) was found in these additional Muslim families. The combined analysis of primary and additional Muslim families retained the peak of linkage at DXS8055 (LOD 1.49; *P* = 0.0045; data not otherwise shown) on Chromosome Xq23.

For refined mapping we corrected [35], [36] for possible type I errors due to over-relatedness of parents by adding inbreeding loops to the pedigrees based on the relationships between parents determined by the PREST analysis (Table 2). Results (data not shown) were essentially similar to the uncorrected data (Tables 3 and 4), indicating that there was no inflation of LOD scores due to consanguinity and over-related parents.

### Analysis of population substructure using STRUCTURE

**Figure 2:**
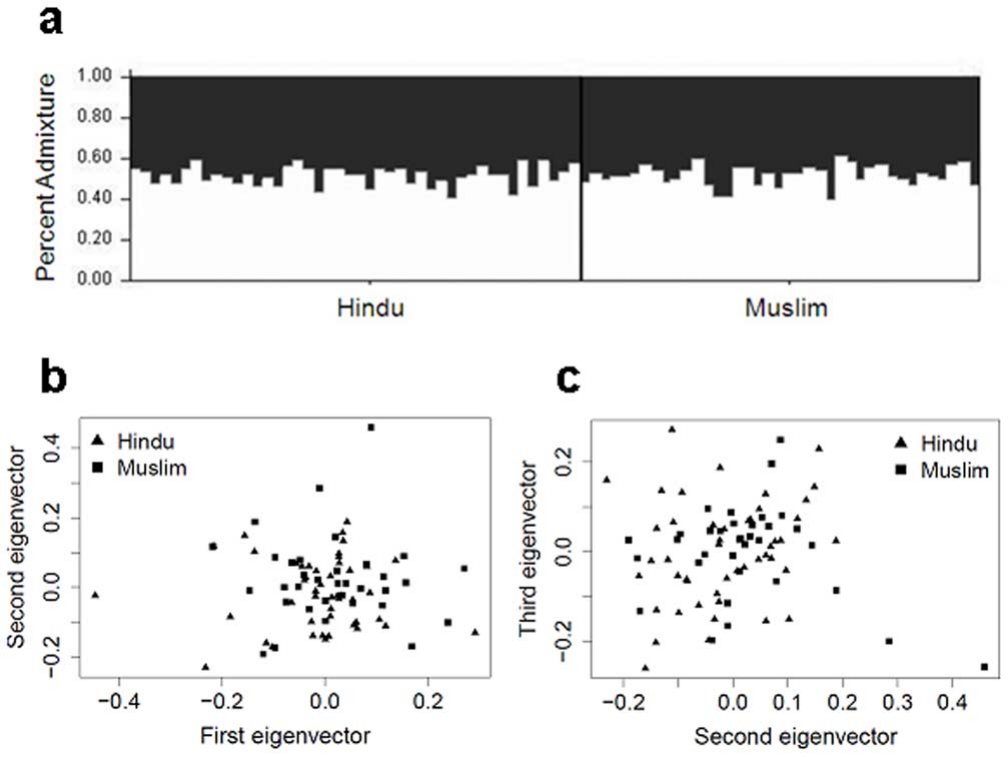
Estimates of population substructure using a subset of unrelated individuals from the primary scan families. (a) Analysis using STRUCTURE for the admixture model assuming correlated frequencies and K = 2 subpopulations. Each individual is represented by a single vertical line, with each black or white segment corresponding to the proportion of the individual assigned to each of the K = 2 pre-defined subpopulations. Plots of (b) the first and second, and (c) the second and third, principal components analyzed using SMARTPCA. The reported religion for each unrelated individual is shown.

Whilst some evidence for linkage peaks defined by religious group was observed in the primary genome scan data, these were not necessarily maintained when a second set of families for each religious group was studied. To determine whether Hindu and Muslim families used in the primary genome scan showed underlying genetic differences, we carried out analysis of population substructure using 480 autosomal primary scan markers and the model-based clustering software STRUCTURE [37]. For all three ancestry models tested, the estimated posterior probabilities provided best fit of the data for K = 1, suggesting that there was no genetic heterogeneity. Indeed, for K = 2 subpopulations, the admixture proportions assigned to each individual were roughly equal (Figure 2a), indicating the absence of population substructure in this Indian cohort. Principal components analysis performed using SMARTPCA also failed to provide any evidence of population substructure associated with religious group in the Indian population studied (Figure 2b and 2c). All of the Muslim family founders fall within the same clusters of Hindu family founders formed when 1^st^ versus 2^nd^ (Figure 2b), 1^st^ versus 3^rd^ (not shown) or 2^nd^ and 3^rd^ (Figure 2c) principal components were plotted.

### CART analysis and spatial interpolation of LOD scores

**Figure 3:**
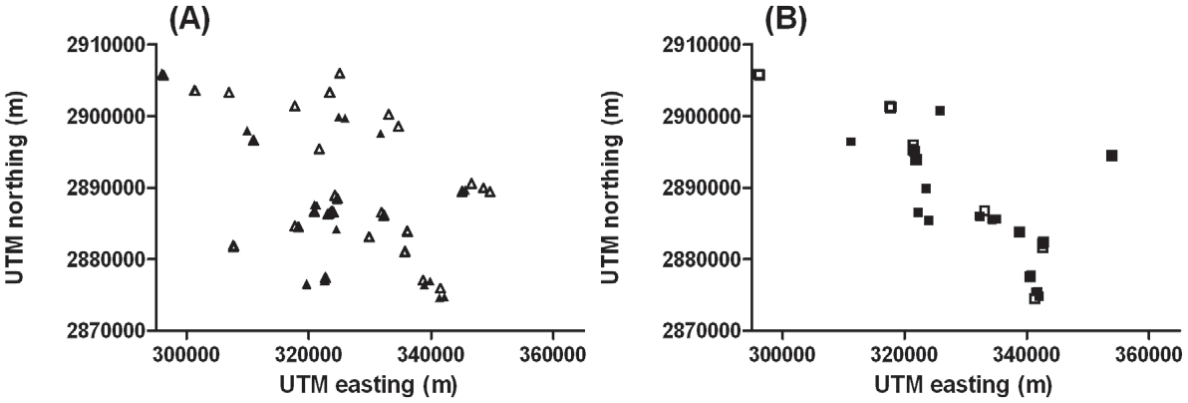
Spatial locations of the (a) Hindu and (b) Muslim primary scan (closed symbols) and additional (open symbols) Indian clinical VL families. The UTM northing and easting values for each family are shown on the vertical and horizontal axis, respectively. The axes indicate distances in meters, thus 58 km east to west and 31 km north to south. The largest distance between two families is approximately 55 km.

**Figure 4:**
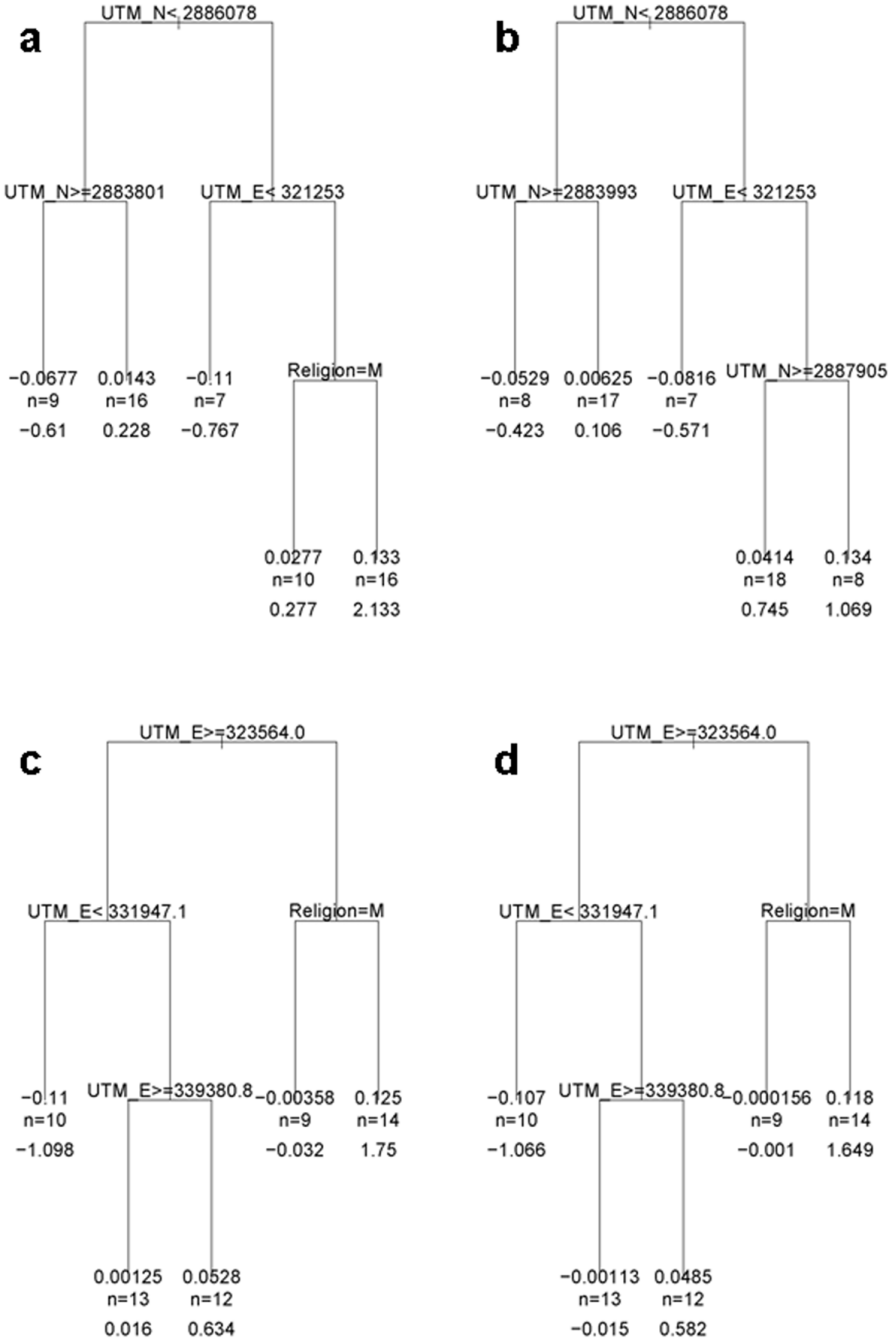
CART trees for markers at the peaks of linkage on Chromosomes 6 and 8: (a) D6S244; (b) D6S1617; (c) D8S516; and (d) D8S520. UTM  =  Universal Transverse Mercator; E =  easting, N =  northing, in meters. M =  Muslim. n is the number of families contributing to the cluster on each branch of the tree; the number directly above is the average LOD score for these families; the number directly beneath is the summed LOD score for these families.

Although analysis of the primary genome scan data by religion identified peaks of linkage on Chromosomes 6 and 8 that appeared to be Hindu-specific, these did not replicate in a second sample from the same general region of Bihar State in India (Figure 3). In addition, STRUCTURE and SMARTPCA provided no evidence for population substructure aligned with religious group. We therefore considered whether geographical clustering of families *per se* might contribute to the different chromosomal linkages to disease susceptibility observed in the primary genome scan data. To evaluate statistically whether geographical location or religion was influencing linkage results, we used CART analysis. For each marker on each chromosome, regression trees were used to explore the relationship of LOD score to the UTM northing and easting locations and to religion. For Chromosome 6, the regression tree for marker D6S244 (Figure 4a) at the original “Hindu” peak of linkage showed that the primary binary decision for families contributing to linkage at this marker was determined by whether they lived north or south of the UTM northing 2,886,078 m. To the right of the tree (Figure 4a; north of UTM northing 2,886,078 m) a second binary decision is based on families living west or east of the UTM easting 321,253 m. Thereafter, the splitting function on the right hand side of the tree (east of UTM easting 321,253 m) is based on religious group, with 16 Hindu families contributing a total LOD score 2.133 (nominal *P* = 0.0009). Hence, there was geographical heterogeneity even amongst the original 31 Hindu families that contributed to the primary linkage peak observed at Chromosome 6p25.3-p24.3. Splitting functions at all other parts of the D6S244 tree were based on UTM northings/eastings rather than religious group. For the adjacent marker D6S1617 (Figure 4b) the ultimate splitting function on the right hand side of the regression tree was based on the UTM northing 2,887,905 m rather than religious group.

For Chromosome 8, the regression tree for marker D8S516 (Figure 4c) at the original “Hindu” peak of linkage showed that the primary binary decision for families contributing to linkage at this marker was determined by whether they lived east or west of the UTM easting 323,564 m. Thereafter, the splitting function on the right hand side of the tree (west of UTM easting 323,564 m) is based on religious group, with 14 Hindu families contributing a total LOD score 1.75 (*P* = 0.002). Following the primary binary decision down the left hand side of the tree (east of UTM easting 323,564 m) reveals a second cluster of 12 families living west of the UTM easting 339,381 m that also contribute a total LOD score 0.634 (nominal *P* = 0.04) for which there is no further splitting function based on religious group. Essentially similar results were obtained for the adjacent marker D8S520 (Figure 4d).

**Figure 5:**
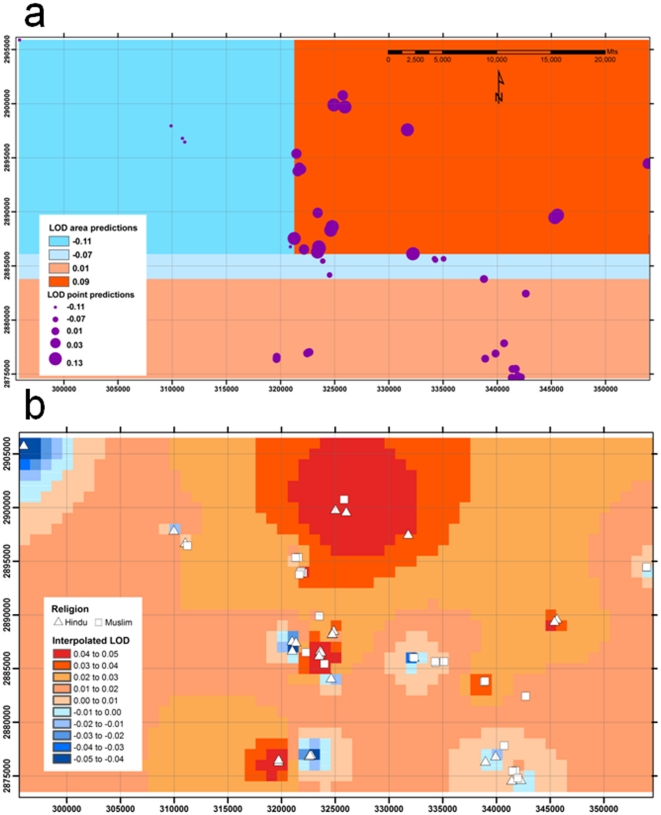
Spatial analyses for markers at the peak of linkage on Chromosome 6: (a) provides a spatial representation of the CART tree for D6S244 presented in **Figure 4a**; (b) shows the spatial interpolation of LOD scores for D6S244 independently derived in ArcGIS. Axes show Universal Transverse Mercator (UTM) eastings (X axis) and northings (Y axis) in meters.

Figure 5a provides spatial representation of the CART analysis for the marker D6S244 at the peak of linkage on Chromosome 6, defining the broad geographical quadrants that contain families that contribute to mean or summed LOD scores at each branch of the tree. Figure 5b shows the more refined result of spatial interpolation used to visualize the geographical distribution of LOD scores for marker D6S244. Comparison of Figures 5a and 5b demonstrate hot spots for LOD scores in the interpolation plots that broadly correspond to the spatial regions defined by the CART analysis. The spatial patterns for CART and the spatial interpolation analysis are similarly matched for marker D8S516 at the peak of linkage on Chromosomes 8 (Figure S1). Overall, CART analysis and spatial interpolation of the primary GWLS data support clustering of families contributing to the linkage peaks on Chromosomes 6 and 8 that has as much to do with geographical location as to do with religious group. This provides some explanation as to why putative “Hindu” peaks on Chromosomes 6 and 8 of linkage did not replicate in the second set of Hindu families (Figure 3) sampled from this broad region of India.

On Chromosome X, primary and refined mapping and replication studies suggested a broad region of linkage to VL that was specific for Muslim families. In the CART analysis, none of the binary decisions and splitting functions at any part of the trees for markers across the broad region of linkage was based on religious group. Instead, for markers DXS6799 and DXS8020 (Figure S2) used in the primary scan (see Table 4), the primary binary decision and splitting function clustered families east of the UTM easting 322,321 m, with 16 families living west of this split contributing to LOD scores of 1.14 and 1.15, respectively. For the more distal markers DXS8055, DXS1001 and DXS8059, the primary binary decision and splitting function clustered families north of the UTM northing 289,824 m, with 16 families north of this split contributing to LOD scores of 1.62, 1.84 and 1.53, respectively (Figure S2). For all of these markers, additional secondary and tertiary clustering was also based on UTM northing/easting locations. Overall, this points to clustering of families contributing to the broad linkage peak on Chromosome X based on geographic heterogeneity rather than religious group. Similar geographic clustering of the additional Muslim families compared to the primary genome scan Muslim families (Figure 3) perhaps accounts for the observed replication at Chromosome X.

Geographic heterogeneity in families contributing to linkage peaks on Chromosomes 2 and 11 were also observed in the CART analysis (Figure S3). For marker D11S1780 at the peak of linkage and adjacent markers, binary decisions and splitting functions at all parts of the trees were based on UTM northing/easting locations rather than religious group (Figure S3b). For marker D2S293 at the peak of linkage on Chromosome 2, religious group contributed to one end point binary decision with 12 Muslim families providing a LOD score of 1.638 split from 16 Hindu families providing a LOD score of 0.285 (Figure S3a). Binary decisions and splitting functions for adjacent markers were all based on UTM northing/easting locations. Again, this heterogeneity provides some explanation as to why the region of linkage on Chromosome 2 failed to replicate in additional families.

## Discussion

Results presented here provide preliminary evidence (≥2 adjacent markers allele-sharing LOD≥0.59; nominal *P*≤0.05) for susceptibility loci for clinical VL in India on Chromosomes 2, 5, 6, 7, 8, 10, 11, 20 and X. Linkage peaks at 2q12.2-q14.1 and 11q13.2-q23.3 were supported by refined mapping across all primary genome scan families, but only 11q13.2-q23.3 replicated in a second set of families. Linkage peaks at 6p25.3-p24.3 and 8p23.1-p21.3 that appeared to be specific to Hindu families, and at Xq21.1-q26.1 putatively specific to Muslim families, were likewise supported by refined mapping in primary genome scan families, but only Xq21.1-q26.1 was replicated across all Muslim families. As with previous GWLS studies in Brazil [15], [16], none of these linkage peaks achieved genome-wide significance as defined by Lander and Kruglyak [18]. Nevertheless, two points are of note. Firstly, none of the regions identified as containing putative susceptibility loci in this Indian study matched any of the regions of linkage identified by GWLS in Sudanese [14], [17] (Chromosomes 1p22, 6q27 and 22q12) or Brazilian [15], [16] (6q27, 9p21 and 17q11-q21) studies. Indeed, thus far only Chromosome 6q27 has been identified as a region of linkage for VL susceptibility across more than one population [15], [17]. Secondly, as with studies carried out in Sudan [14], [17], this Indian study provides evidence for local geographic clustering of families that segregate for susceptibility alleles at loci mapping to different chromosomal regions. In Sudan [17], this was attributed to founder effect and consanguinity in villages recently established (<30 years) by a small number of immigrant families, between which little intermarriage had yet taken place. In India, we hypothesized initially that heterogeneity might relate to lack of intermarriage between families belonging to different religious groups. In a previous study Bhattacharyya et al. [54] noted negligible male gene flow across ethnic boundaries in India as revealed by Y chromosome haplotype analysis while there is detectable occurrence of Middle Eastern paternal lineages in contemporary Indian Muslims [49], [50]. Nonetheless, most findings suggest that the Muslim populations of India have primarily derived from cultural conversion and thus are closely related to their geographically neighboring non-Muslim populations [49], [50]. Consistent with these reports, there was no fundamental genetic substructure that aligned with religious group in our study population. We thus conclude that the geographical heterogeneity observed in India might relate (a) to local clustering of families in which there is a high rate of consanguinity, and/or (b) to limited intermarriage across villages located far apart within the study area. In some cases this might be a cluster of families belonging to the same religious group, but religious group *per se* is not the genetic reason for linkage peaks that appeared at first to be Hindu- or Muslim-specific. These results expand on previous findings by Gutala et al (2006) showing greater correlations between genetics and geography as opposed to religion when comparing populations from North and South India. Other variables related to the complexity of Indian populations, such as caste, socio-economic status, and environmental risk factors related to VL infection [55] could further affect the action (e.g. in a gene x environment interaction) of different susceptibility genes between clusters of families. Our use of spatial analyses provides the potential for integrated analysis of genetic and environmental risk factors as a continuing focus of epidemiological studies designed to determine the major risk factors for disease in India.

The fact that different linkage peaks are identified within and between the major global foci on VL in India, Sudan and Brazil is not necessarily surprising, since the complex nature of the disease means that we expect many loci to contribute small effects to disease susceptibility. The species (*L. donovani* in India and Sudan vs. *L. infantum chagasi* in Brazil) or strain of the parasite might also determine different genetic control. Ability to map these loci has been limited by employment to date [14]–[17] of sample sizes and linkage analyses that have been powered to identify single major genes or, at best, oligogenic control. Large-scale genome-wide association studies currently being undertaken as part of phase 2 of the Wellcome Trust Case Control Consortium (WTCCC2) may help to redress this. However, failure to replicate linkage peaks between and within global foci of VL does not discount the possibility that the novel regions of linkage identified in this Indian GWLS contain interesting candidate genes for which relevance to host immune defenses against *Leishmania* infection has been demonstrated in humans and mice. For example, the interleukin (IL)-1 cytokine family gene cluster which plays a prominent role in pro-inflammatory responses to infection lies close to the peak of linkage on Chromosome 2q12.2-q14.1. Lipopolysaccharide (LPS)-induced IL-1β production is inhibited in human peripheral blood monocytes (PBMCs) infected with *L. donovani* [56], [57], while the *Leishmania* lipophosphoglycan (LPG) mediates its suppressive effect on IL-1β transcription via a unique promoter sequence [58]. The role of IL-1 levels in disease pathogenesis is also demonstrated in susceptible BALB/c mice [59]. Candidates at 11q13.2-q23.3 include the genes encoding IL-18 and its binding protein (IL-18BP), which regulate interferon-γ induction. During *Leishmania* infection, IL-18 has been shown to act as an important stimulator of Th1 cytokine responses [60], [61], while it can also promote Th2 differentiation depending on the host genetic background and cytokine environment [62], [63]. The peak of linkage on 6p25.3-p24.3 harbours the interferon regulatory factor (IRF) 4, the up-regulation of which mediates cytokine-driven innate and Th1 immune responses [64], [65]. IRF-4 is involved in T cell differentiation towards the Th1 and Th2 lineages [66] and its protective effect against apoptosis, shown in *IRF-4*-deficient C57BL/6 mice infected with *L. major* [67], is of specific interest to VL susceptibility. Genes encoding a number of defensins, with microbicidal and cytotoxic effects against bacterial and fungi infections, are potential candidates at 8p23.1-p21.3. The IL-13 receptors alpha-1 (*IL-13RA1*) and -2 (*IL-13RA2*), and the NF-κB repressing factor (*NKRF*) and activating protein (*NKAP*) are also under the extended peak at Xq21.1-q26.1. Expression of the IL-13 alpha-2 receptor is required for activation of the TGF-β1 promoter in macrophages [68], which could be the pathway via which IL-13 exerts its negative effects during *Leishmania* infection [69]. Parasite survival within macrophages could be mediated through NKRF's role in inhibiting production of the inducible nitric oxide synthase (iNOS) and subsequent generation of nitric oxide [70]. In contrast, NKAP-activated NF-κB promotes Th1 responses in *L. major* infection [71], while C57BL/6 mice knocked-out for *NF-κB_1_* [72] and *NF-κB_2_* [73] develop chronic unresolving lesions associated with persistent parasites.

In summary, results of the GWLS presented here point to novel loci that may contribute to susceptibility to VL in India. Further genetic and functional characterization of some of these genes in relation to VL is in progress, results of which will contribute to our understanding of the pathogenesis of this important and complex infectious disease.

## Supporting Information

Figure S1 **Spatial analyses for markers at the peak of linkage on Chromosome 8: (a) provides a spatial representation of the CART tree for D8S516 presented in** **Figure 4c** **; (b) shows the spatial interpolation of LOD scores for D8S516 independently derived in ArcGIS.** Axes show UTM eastings (X axis) and northings (Y axis) in metres.
(TIF)

Figure S2 **CART trees for markers at the peaks of linkage on Chromosome X: (a) DXS6799; (b) DXS8020; (c) DXS8055; (d) DXS1001; and (e) DXS8059.** UTM  =  Universal Transverse Mercator; E =  easting, N =  northing, in meters. M =  Muslim. N (n) is the number of families contributing to the cluster on each branch of the tree; the number directly above is the average LOD score for these families; the number directly beneath is the summed LOD score for these families.
(TIF)

Figure S3 **CART trees for markers at the peaks of linkage on Chromosomes 2 and 1: (a) D2S293; and (b) D11S1780.** UTM  =  Universal Transverse Mercator; E =  easting, N =  northing, in meters. M =  Muslim. N (n) is the number of families contributing to the cluster on each branch of the tree; the number directly above is the average LOD score for these families; the number directly beneath is the summed LOD score for these families.
(TIF)
