# Supplementary figures and images for: Classification and Regression Tree and Spatial Analyses Reveal Geographic Heterogeneity in Genome Wide Linkage Study of Indian Visceral Leishmaniasis
Source: PLoS One. 2010 Dec 31;5(12):e15807. doi: 10.1371/journal.pone.0015807 (PMC3013125; doi:10.1371/journal.pone.0015807)

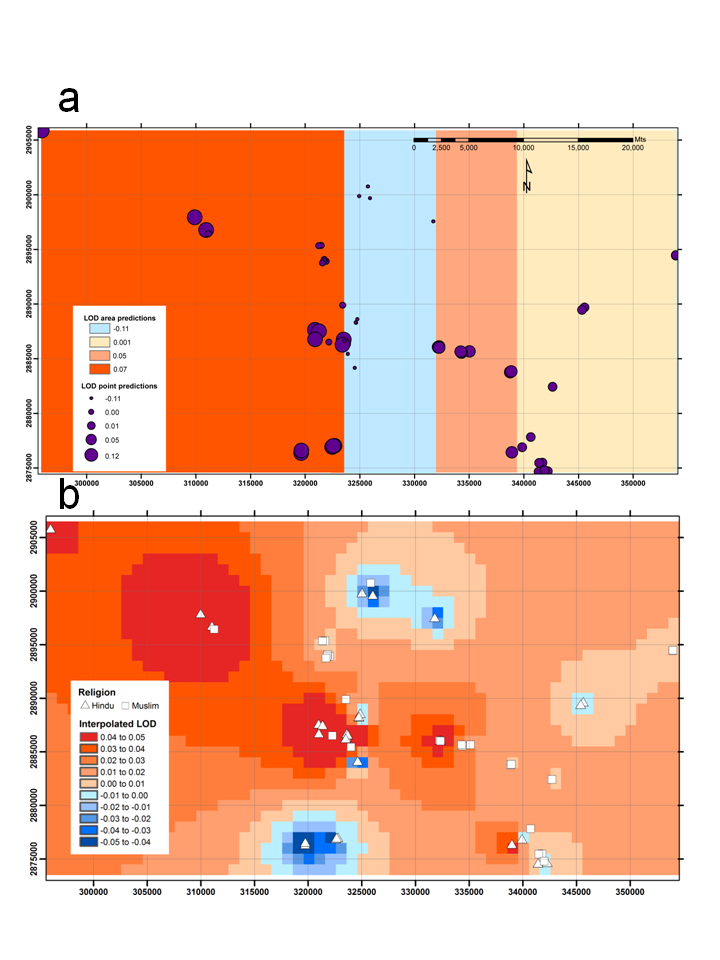

Supplement: Figure S1 — Spatial analyses for markers at the peak of linkage on Chromosome 8: (a) provides a spatial representation of the CART tree for D8S516 presented in Figure 4c ; (b) shows the spatial interpolation of LOD scores for D8S516 independently derived in ArcGIS. Axes show UTM eastings (X axis) and northings (Y axis) in metres. (TIF) [file pone.0015807.s001.tif]

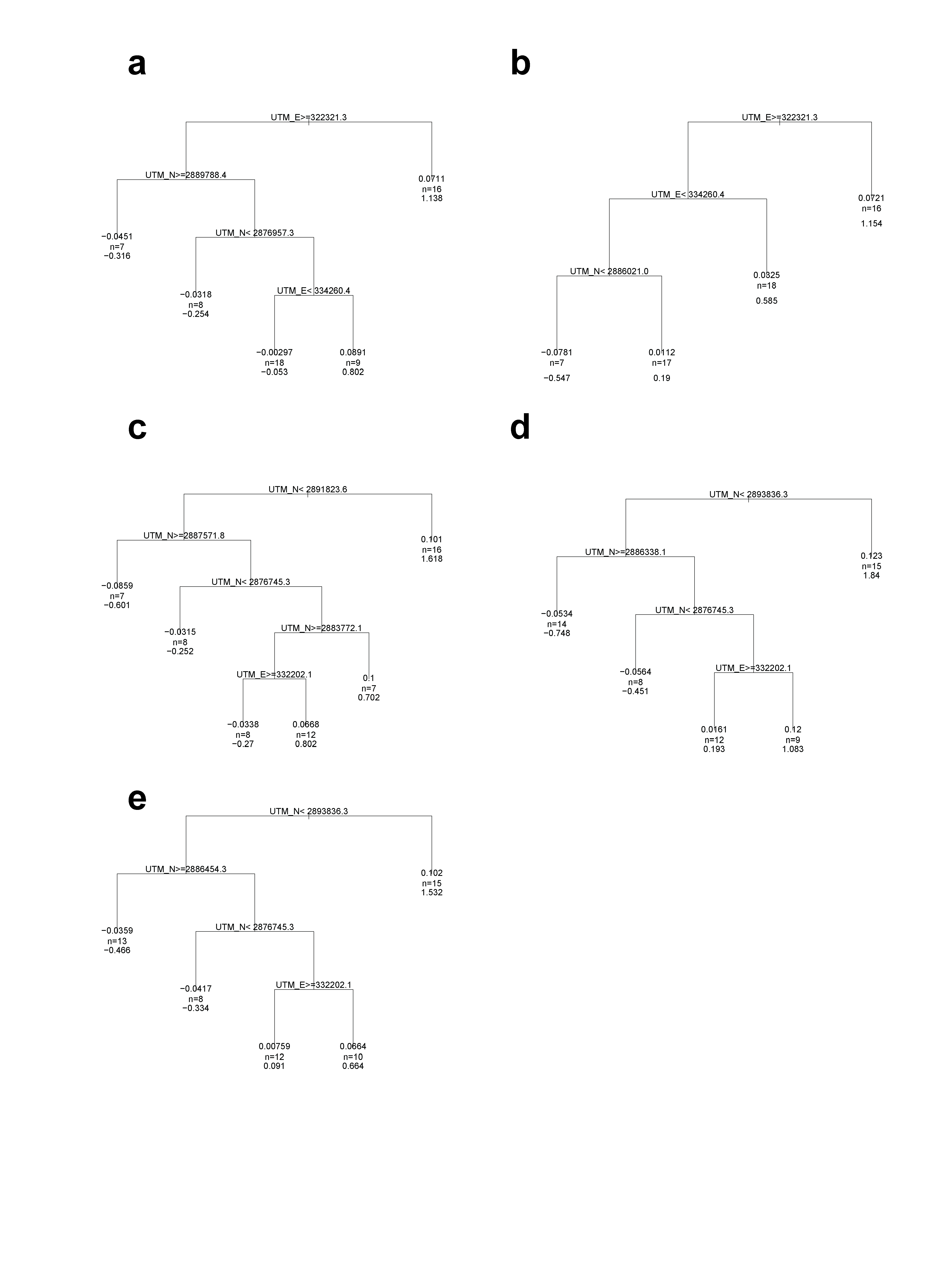

Supplement: Figure S2 — CART trees for markers at the peaks of linkage on Chromosome X: (a) DXS6799; (b) DXS8020; (c) DXS8055; (d) DXS1001; and (e) DXS8059. UTM = Universal Transverse Mercator; E = easting, N = northing, in meters. M = Muslim. N (n) is the number of families contributing to the cluster on each branch of the tree; the number directly above is the average LOD score for these families; the number directly beneath is the summed LOD score for these families. (TIF) [file pone.0015807.s002.tif]

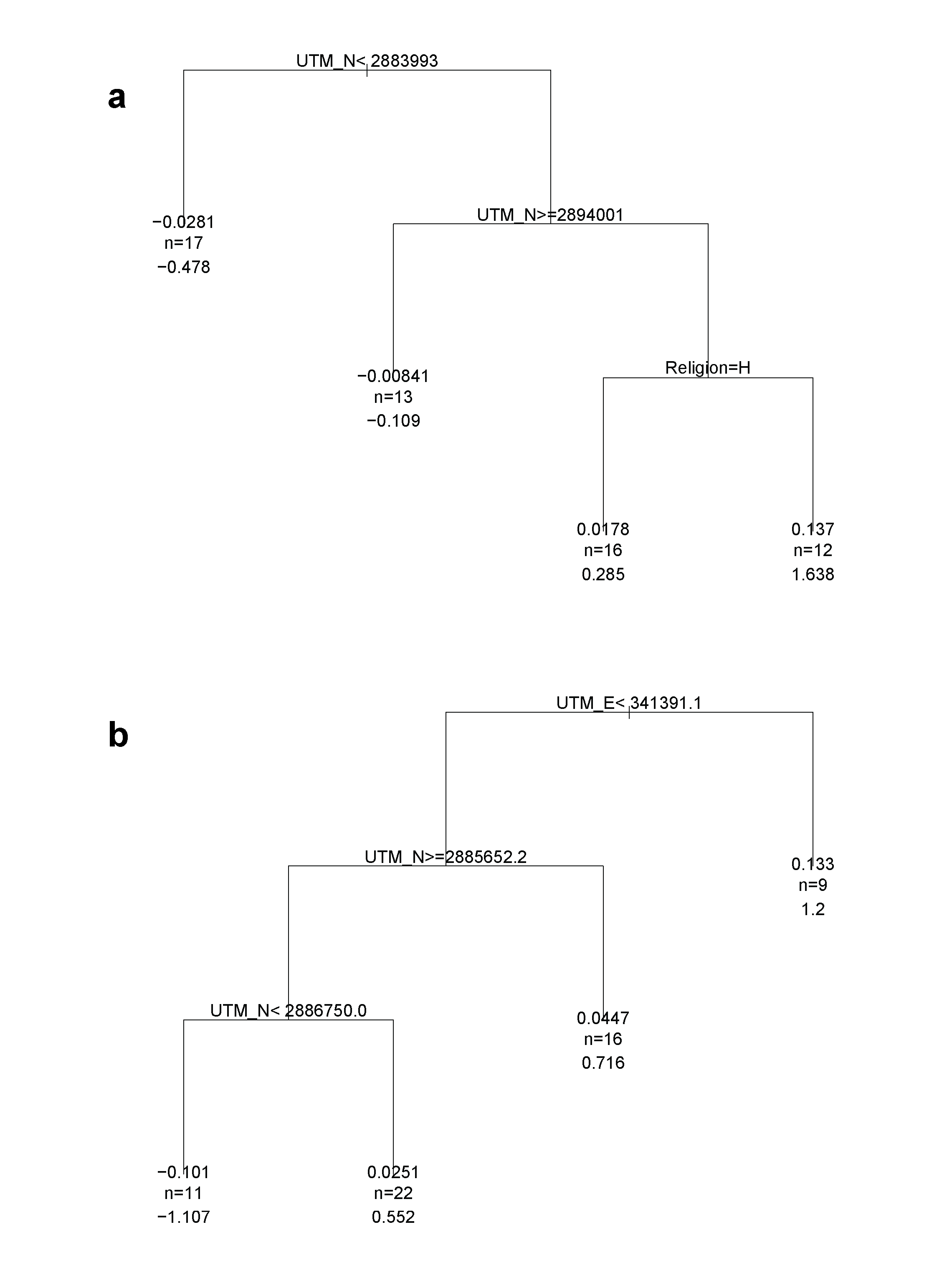

Supplement: Figure S3 — CART trees for markers at the peaks of linkage on Chromosomes 2 and 1: (a) D2S293; and (b) D11S1780. UTM = Universal Transverse Mercator; E = easting, N = northing, in meters. M = Muslim. N (n) is the number of families contributing to the cluster on each branch of the tree; the number directly above is the average LOD score for these families; the number directly beneath is the summed LOD score for these families. (TIF) [file pone.0015807.s003.tif]
